# The Effect of Wind on the Rate of Heat Loss from Avian Cup-Shaped Nests

**DOI:** 10.1371/journal.pone.0032252

**Published:** 2012-02-28

**Authors:** Caragh B. Heenan, Roger S. Seymour

**Affiliations:** Department of Ecology, Evolution and Landscape Science, The University of Adelaide, Adelaide, South Australia, Australia; University of Regina, Canada

## Abstract

Forced convection can significantly influence the heat loss from birds and their offspring but effects may be reduced by using sheltered micro-sites such as cavities or constructing nests. The structural and thermal properties of the nests of two species, the spiny-cheeked honeyeater (*Acanthagenys rufogularis*) and yellow-throated miner (*Manorina flavigula*), were measured in relation to three wind speeds. Nest dimensions differ between the two species, despite the similar body mass of the incubating adults, however nest conductance is comparable. As wind speed increases, so does the rate of heat loss from the nests of both species, and further still during incubation recesses. The significance of forced convection through the nest is a near-doubling in heat production required by the parent, even when incubating at relatively low wind speeds. This provides confirmation that selecting a sheltered nest site is important for avian reproductive success.

## Introduction

The microclimate properties that are critical to adult birds include wind, radiation, air temperature and humidity, and these directly affect the thermoregulatory demands with which a bird must cope [1]–[3]. Wind, which is forced convection (henceforth referred to as convection), is often considered to be more important for heat loss than conduction and evaporation [3]–[5]. The energetics of a variety of avian species exposed to wind has been explored using doubly-labelled water, time energy budgets and respirometry techniques in wind tunnels [6]–[9]. Webster and Weathers [10] found that heat production in verdins (*Auriparus flaviceps*) can rise by nearly 30% when wind speed increases from 1.8 to 10.8 km h^−1^, whereas Tracy [11] argued that heat loss may vary by up to 100% for some individuals under different wind speed conditions.

Energy demands of thermostasis may be greatest when roosting or during reproduction [1], [12], [13]. Therefore, it is expected that there would be strong selective pressure for birds to minimise thermoregulatory stresses to the individual and offspring. Reducing air movement over birds moderates their convective heat loss and this can be achieved by using sheltered micro sites such as cavities and domed nests [13], [14]. Of primary interest here is how convection can influence heat loss from the nest of reproducing individuals, as the rate of heat loss can influence the outcome of a breeding attempt and consequently lifetime reproductive success [12], [15]. Reproduction in birds via oviparity necessitates developmental conditions to be modulated externally, provided by the reproducing birds through modification of their own metabolism [16], [17]. While the energetics of birds under different wind regimes has only been investigated in non-incubating individuals, it is expected that wind would also increase the rate of heat loss for reproducing birds. Appropriate nest site selection can reduce heat loss through convection, however such savings may be small compared to those produced by the addition of an insulating nest [1], [18]. Consequently, nests might be expected to be shaped by selection over evolutionary time to approach functional optima and reflect the microclimate conditions to which birds are exposed, including convection [16].

Nest structure and placement has been reported widely in the literature in recent years, as the importance of such structures has become more apparent [reviewed by 19]. Methods for determining the effect of wind on heat loss from the nest largely consist of either heat loss modelling [4] or nest orientation correlations [20], [21]. Nest orientation is expected to change the nest microenvironment due to the effects of wind and thus sparrow (*Ammodramus savannarum* and *Aimophila aestivalis*) and meadowlark (*Sturnella magna*) nests are primarily oriented away from prevailing winds [22], [23]. Woodpecker (Picidae) nest orientations provide shelter from the wind and rain [24], while nest orientation for a variety of avian species is correlated with modified nest temperatures [25]–[30].

There may be differences in nest structure that contribute to reductions in convective heat loss [31], [32]. Palmgren and Palmgren [33] were the first to assess the influence of convection on nest insulation and found that heat loss increases in turbulent conditions by 44% in the common rosefinch (*Carpodactus erythrinus*) to 91% in the chaffinch (*Fringilla coelebs*). Kern [31] found that elevated nests of the white-crowned sparrow (*Zonotrichia leucophrys*) were better insulated than ground nests and proposed that the increased insulation may offset the increased convective cooling to which they are exposed. Pectoral sandpiper (*Calidris melanotos*) nest structure is an example of how a compromise can be found between multiple unfavourable variables [16]. While deep nest scrapes would reduce convective heat loss from eggs, the eggs would in turn experience cooler ground temperatures. Scrapes are therefore constructed such that the eggs are positioned at an optimal depth for minimising forced convection as well as the rate of heat loss to the substrate. The choice of materials used for nest construction may also be partly driven by the need to reduce convective heat loss [34].

There is a paucity in the knowledge regarding the effect of wind on the insulative properties of exposed nests and how wind may influence the heat loss from the clutch, and in turn, the energetics of the incubating parent. Our earlier study on the thermal properties of nests involved conductive heat loss through whole nests in essentially still air [35]. The results from 36 species of birds with body masses ranging from 8 to 360 g demonstrated that cup-shaped nests were constructed primarily for support rather than insulation. Insulation was evaluated by measuring its inverse, thermal conductance. This is the rate of heat flux (watts) moving across the nest wall, per degree of temperature difference between the inside and outside of the nest, based on Newton's Law of Cooling [11], [36]. Well-insulated nests have a low conductance and vice versa. The present study is designed to investigate the role of convective heat transfer through cup-shaped nests of two species with similar body masses, but of different nest construction, thin versus thick walls. Here, the thermal conductance is again measured, but within a wind-tunnel. In the present study, the ‘effective’ thermal conductance and ‘effective’ material conductivity is measured (henceforth referred to as conductance and conductivity, respectively), as both thermal properties in this context include the effects of conductive and convective heat flow. If nest wall structure is important in preventing heat loss via convection we expect that there would be differences in nest conductance that are related to the thickness of the nest wall or the conductivity of the nest material in windy conditions. Furthermore, we compare the heat loss from nests where the opening is sealed with a Styrofoam lid to those without a lid. This was conducted to reflect differences in heat loss from attended and unattended nests.

## Methods

Nests were borrowed from the South Australian Museum ornithology collection and were selected for measurement if they were in a good condition and had no branches obscuring the opening of the nest. Nests that were damaged were excluded from analyses. Nests were collected between 1976 and 1992, with collection information missing for five out of the 15 nests. For those where collection date was known, there was no significant difference in the year the nest was collected when comparing the two species (*X^2^* = 1.86, DF = 1, *P* = 0.17). All nests were from museum collections, stored in a similar way, which should have reduced any bias resulting from degradation and storage. Notes on the construction of each nest were made; including condition, attachment, materials, nest shape and weave density.

### (a) Study species

**Figure 1:**
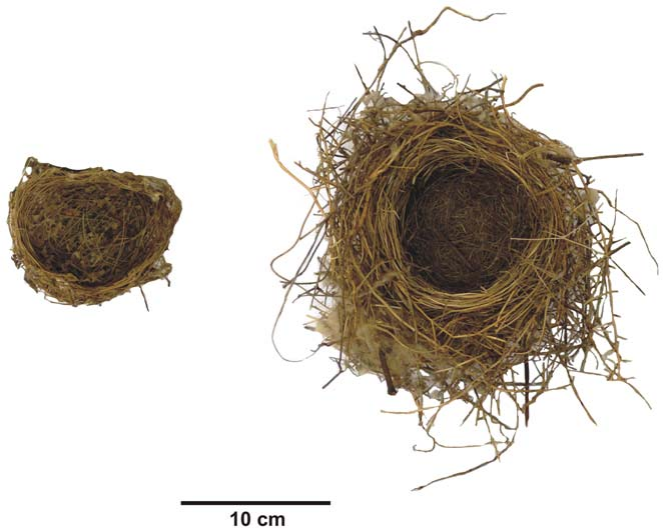
A nest of *Acanthagenys rufogularis* and *Manorina flavigula*. The nest of *A. rufogularis* (left) is smaller and has thinner nest walls than *M. flavigula* (right) but a greater nest wall density.

A total of eight spiny-cheeked honeyeater (*Acanthagenys rufogularis* Gould, 1838) and seven yellow-throated miner (*Manorina flavigula* Gould, 1840) nests were measured. The two species were selected as they both have similar parent masses (50 g and 55 g, respectively), however different nest structure (Figure 1). While *M. flavigula* nests have larger dimensions, *A. rufogularis* constructs a nest with a denser wall compared to *M. flavigula*. In addition, the nest dimensions for *A. rufogularis* are lower than what would be predicted for a bird of this size, while nests of *M. flavigula* are larger than predicted [35].

The two species have largely overlapping geographical distributions throughout Australia and breed in similar habitats, usually open woodland or shrubland in arid and semi-arid zones [37]. Both species are capable of breeding year-round but most of the breeding occurs between June/July through to March. While *A. rufogularis* tends to construct their nests suspended in the top or outer edge of plant canopies (1 to 3 m above ground), *M. flavigula* nests are regularly found in dense canopy close to the trunk of the plant (4 to 5 m above ground).

### (b) Nest dimensions

**Figure 2:**
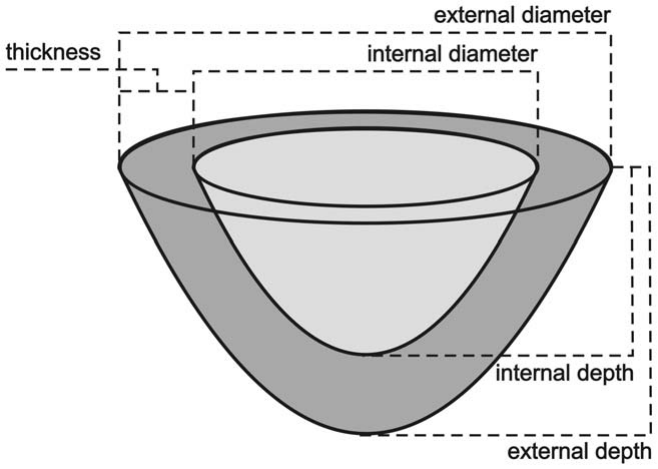
A nest diagram showing the dimensions used to calculate surface area. Both internal (light grey) and external (dark grey) nest surface area were calculated to obtain the geometric mean surface area.

The physical dimensions of the nests were measured with callipers and a micrometer to the nearest millimetre, including the nest thickness (X, Figure 2), internal and external diameter (d) and height (h). Those nests without supporting structures attached were weighed on a Mettler digital analytical balance (model AE163, Zürich, Switzerland).

The internal and external nest surface area (A) was approximated using the equation for half of a prolate spheroid (Equation 1 of Heenan & Seymour [35]), using values for internal and external diameters and heights. The average surface area (Ā) was calculated as the geometric mean of the internal and external surface area. Nest volume was calculated as half of the volume for a prolate spheroid based on external dimensions, minus half of the volume of a prolate spheroid based on internal dimensions (Equation 1 of Heenan, Paton & Seymour [38], in Preparation). The density of the nest was calculated for each species. Nest density (ρ, g cm^−3^) was calculated as the nest mass (M_N_) divided by the nest volume (V_N_).

### (c) Wind tunnel

**Figure 3:**
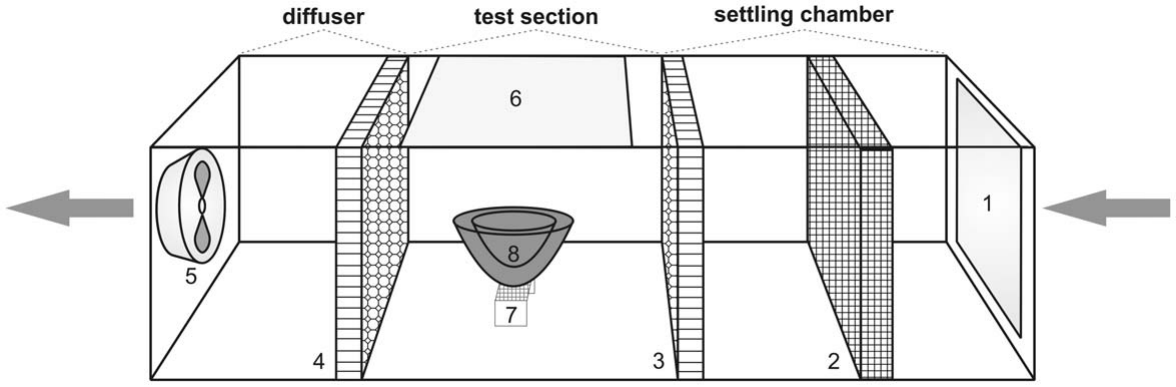
The wind tunnel used to alter experimental wind conditions. Sections include the settling chamber, test section and diffuser. Wind flows in direction of arrows. 1. Wind tunnel air inlet; 2. Wind filter; 3. Inlet honeycomb screen; 4. Outlet honeycomb screen; 5. Axial fan; 6. Test section access door; 7. Mesh base; 8. Nest. Not drawn to scale.

A wind tunnel was constructed to enable wind speed surrounding the nest to be controlled and measured (Figure 3). The wind tunnel consisted of a long cardboard box (35.5 cm width; 36.5 height; 139 cm length), divided into three main chambers: the settling chamber, the test section and the diffuser. The settling chamber consisted of a filter made from a double-layer fly-wire mesh screen. This was used to prevent large airborne particles from entering the chamber and causing undesirable turbulence in the flow [39], [40]. A 4 cm wide honeycomb screen made from plastic straws (1 cm in diameter), glued together in alternating layers, was placed towards the end of the settling chamber. The honeycomb screen acts to straighten the flow, reducing turbulence, as well as eliminating the cross-flow component [39], [41]. A second honeycomb screen was placed at the beginning of the diffuser.

The test section was situated between the two honeycomb screens, with access provided by an outward-opening door. The door was sealed closed during the experiments with metal corner brackets clipped in place with Velcro.

A duct mounted axial fan (ø150 mm, model MT132, Fantech, Dandenong, Australia) was placed at the outlet end to suck wind through the tunnel. The speed of the fan was manipulated with a variable voltage transformer (model Voltac SB-5 IKVA, Yokoyama Electric Co. Ltd., Japan) by varying the voltage from 0 to 240 V. The maximum wind speed achievable was 3.4 km h^−1^.

Wind speed was measured with a hot-wire anemometer (model AM-4204HA, Lutron Electronic, Coopersburg, USA) at radial interval positions through the centre of the test section when the fan was at its maximal speed. This was to ensure that wind speed was relatively consistent at all points in the chamber and that differences in measurements would not result from slight changes in nest placement. Wind speed varied slightly throughout the chamber test section, ranging from 2.7 to 3.1 km h^−1^ (±7%) during the test runs. The anemometer was also turned sideways and moved throughout the chamber to confirm that movement of air through the tunnel was laminar and did not consist of turbulent flow or produce a cross-flow component.

The nest was placed in the centre of the test-section on a 4 cm high wire-mesh strip to allow for free air-flow around it. The cross-sectional area of the wind tunnel blocked by the nest ranged from to 2–9% (mean 5±1%). The anemometer was suspended above the lid of the nest to measure the wind-speed, which was recorded for each nest and treatment. Nest measurements were repeated at three wind speeds: 0, 0.8 and 3.1 km h^−1^. These speeds are described in this paper as ‘still’, ‘calm’ and ‘light air’, respectively, according to Beaufort scale definitions in Allaby [42].

We tried to maintain a consistent wind speed by setting the variable voltage regulator to the same output each time, the average speed detected (Table 1) surrounding the open nests differed for each species for the light air treatment (*X^2^* = 5.53, DF = 1, *P* = 0.019) but not within the calm (*X^2^* = 0.13, DF = 1, *P* = 0.72) treatment. There was no significant difference in the wind speed detected around sealed nests for each species within both the calm (*X^2^* = 1.64, DF = 1, *P* = 0.20) and the light air treatment (*X^2^* = 3.16, DF = 1, *P* = 0.075). Wind speed in the still air treatments was consistently 0 km h^−1^.

**Table 1:** Comparison of wind speeds between open and sealed treatments for nests of *Acanthagenys rufogularis* and *Manorina flavigula*.

		Wind speed (km h^−1^)	
Species	Treatment	Calm	Light air
***Acanthagenys rufogularis***	**Open**	0.76±0.04	3.03±0.10
	**Sealed**	0.71±0.02	3.06±0.04
***Manorina flavigula***	**Open**	0.77±0.04	3.19±0.08
	**Sealed**	0.74±0.04	3.14±0.07

Statistics include the mean ± 95% confidence interval.

The anemometer was inserted into the nest space through a hole in the nest lid to determine the proportion of wind passing through the nest wall under light air conditions. The wind detected in the nest cavity of *A. rufogularis* was 0.6±0.1 km h^−1^ and for *M. flavigula* the nest cavity wind speed was 0.8±0.1 km h^−1^. The ratio of the internal nest wind speed to tunnel wind speed was then compared for the two species. The ratio for nest∶tunnel wind speed was 0.2±0.1 for *A. rufogularis* and 0.3±0.1 for *M. flavigula*, with no significant difference between the two species (F_1,13_ = 0.65, *P* = 0.44). This was repeated for four nest orientations (nest ID tag facing the front, left, back and right side) along the horizontal axis of rotation. There was no significant difference in the ratio of wind entering the nest cavity to that in the tunnel, irrespective of nest orientation (F_1,13_ = 2.00, *P* = 0.18).

### (d) Total nest conductance

The total thermal conductance (G, mW °C^−1^) of the nests was measured by placing an artificial heat source inside the nest and measuring the heat flow out through the wall (Φ), in relation to the temperature difference across the wall (ΔT). The methods of Heenan & Seymour [35] were used where applicable, with exceptions outlined below. All measurements were carried out in a 10°C constant temperature room.

**Figure 4:**
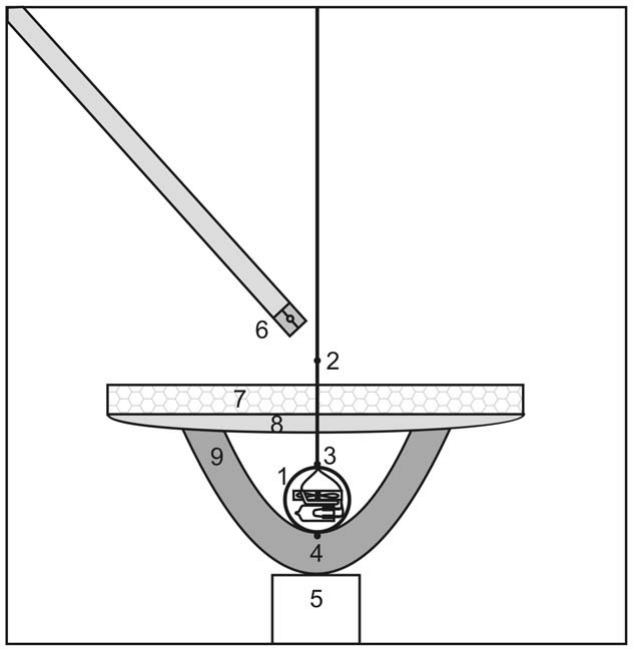
The equipment used to measure nest thermal conductance. 1. Egg heater – consisting of a globe, temperature sensor and fan in an aluminium 3003 alloy shell; 2. External thermocouple; 3. Egg top thermocouple; 4. Egg bottom thermocouple; 5. Mesh base; 6. Anemometer; 7. Styrofoam lid; 8. Cotton padding; 9. Nest. Not drawn to scale.

An artificial egg heater consisted of a 3.35 mm thick aluminium sphere with a diameter of 3.8 cm (3003 alloy, Sharpe Products Inc., New Berlin, USA), within which the heating equipment was placed (Figure 4). The egg was heated internally to 40°C with a 20 W (12 V, 1.67 A) globe (Mirabella International, Tullamarine, Australia). The heated air within the sphere was circulated with a 1.6 cm (3.3 V) fan (Copal Electronics, Tokyo, Japan), mounted above the heat source. The temperature inside the sphere was measured with a LM35DZ temperature sensor and the power supplied to the globe was varied to achieve constant temperature [35]. The opening of the nest was insulated with a 23 mm thick layer of Styrofoam and cotton padding sealed the lip against air leaks.

The voltage supplied to the globe was recorded in a chart application using AD Instruments Powerlab (model ML750, Castle Hill, Australia) and the current sent through the globe was measured with a multimeter (model QM1538, Digitech). The power used to operate the egg fan (Φ_FAN_) is released as heat and this is added to the power requirement of the globe (Φ_GLOBE_). The total power (Φ) required to keep a stable nest temperature was calculated by multiplying the voltage and current from the globe and adding this to the product of voltage and current from the fan, according to Equation 2 of Heenan & Seymour [35].

Two copper-constantan thermocouples ensheathed in polyethylene tubing were placed on the surface of the egg heater, one at the top and one at the bottom, between the egg heater and nest interface. A third thermocouple was placed outside the nest to measure the wind tunnel temperature. The temperature of the wind tunnel fluctuated slightly, within and between treatments, ranging from 10.8 to 12.3°C (11.4±0.1) for the closed treatments and 10.5 to 15.2°C (11.6±0.2) for the open treatments. Temperatures were logged on a portable data logger (model OM-SQ2020-IF8, Omega, Stamford, USA). Thermocouples were calibrated prior to the study by placing them into water of four different temperatures and plotting the thermocouple temperature reading against the temperature reading from a precision calibrated mercury-in-glass thermometer. The resulting calibration regression was used to correct the thermocouple readings.

The equipment was set up to heat the nest for each treatment, followed by an equilibration period of between 30 and 45 min. Measurements were obtained once the heat production rate had stabilised.

The temperature gradient used for calculations was that between the average of the egg heater surface temperatures (T_egg_) and the wind tunnel air temperature (T_a_). Using the temperature gradient and the power required to keep a stable nest temperature, the total thermal conductance of the system (G_TOT_, mW °C^−1^) was calculated using Equation 1.
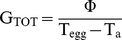
(1)Where the symbols are:

G_TOT_ = System thermal conductance (mW °C^−1^)

Φ = Heat production rate (mW)

T_egg_ = Egg heater surface temperature (°C)

T_a_ = Ambient temperature (°C)

The total conductance of the system includes the conductance of the nest, air within the nest and the Styrofoam lid conductance. The surface area (A_LID_), surface-specific conductance of the lid (G_A-LID_) was calculated according to Heenan and Seymour [35]. For each nest, the surface-specific lid conductance was multiplied by the surface area of the nest opening to obtain the total lid conductance (G_LID_) for each nest. This was subtracted from the total conductance of the nest system to obtain a value representing the conductance from the egg heater surface to the outside of the nest, from here on referred to as total conductance.

This method was repeated for *A. rufogularis* and *M. flavigula* nests without a lid for the calm and light air wind treatments to determine the effect of wind on heat loss from eggs during incubation recesses. To determine the influence of the nest in general, the egg heater was measured in the chamber without the protection of a nest.

### (e) Surface-specific nest conductance

The surface-specific conductance (G_A_, W °C^−1^ m^−2^) was calculated by dividing the total conductance by the geometric mean surface area of the nest.

### (f) Thermal conductivity

The thermal conductivity (k, mW °C^−1^ m^−1^) of the nest material was calculated using the surface area and thickness of the nest, according to Equation 2.
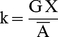
(2)Where the symbols are:

k = Material thermal conductivity (mW °C^−1^ m^−1^)

G = Nest thermal conductance (mW °C^−1^)

X = Nest wall thickness (m)

Ā = Geometric mean of the internal and external surface area (m)

### (g) Statistical analyses

Statistical analyses were performed in JMP IN (SAS Institute Inc., version 4.0.4). Data that met the assumptions required for parametric tests, including normality (Shapiro-Wilk W test) and equal variance (O'Briens test), were subjected to an analysis of variance (ANOVA). Data that did not meet the assumptions required for parametric tests were analysed using the Wilcoxon/Kruskal-Wallis Chi-squared test. Comparisons of species mean values within treatments were made using post-hoc Tukey-Kramer HSD.

An indicator species analysis was performed in PC-Ord (MjM Software, version 5.0) to test whether the materials used in nest construction differed between the two species. The analysis was based on indicator values (percent of perfect indication, based on combining relative abundance values and relative frequency) and a Monte Carlo test of significance of the observed maximum indicator value for materials found in *A. rufogularis* and *M. flavigula* nests.

An alpha value of 0.05 was used for all analyses. Data are expressed as mean ±95% confidence interval.

## Results

Nest dimensions differed between *A. rufogularis* and *M. flavigula*, despite the similar body mass of the incubating adults (Table 2). *M. flavigula* had a greater nest mass, greater nest volume, thicker nest wall, greater surface area, as well as greater diameter and height (both internal and external). However, *A. rufogularis* constructed a nest with a denser nest wall compared to *M. flavigula*.

**Table 2:** Nest dimensions for cup-shaped nests of *Acanthagenys rufogularis* and *Manorina flavigula*.

Dimension	*Acanthagenys rufogularis*	*Manorina flavigula*	Test statistic	*P*-value
**Nest mass (M_N_, g)**	10.30±1.48	41.46±7.53	25.93∧	0.0009*
**Nest volume (V_N_, cm^3^)**	105.09±27.24	745.61±125.83	10.50^#^	0.0012*
**Nest density (ρ, g cm^−3^)**	0.12±0.07	0.06±0.01	9.67∧	0.014*
**Nest thickness (X, cm)**	0.93±0.19	2.96±0.38	96.39∧	<0.0001*
**Surface area (Ā, cm^2^)**	122.46±12.08	233.01±16.82	113.64∧	<0.0001*
**Internal diameter (d_I_, cm)**	7.48±0.34	9.10±0.46	32.18∧	<0.0001*
**External diameter (d_E_, cm)**	9.09±0.59	15.37±1.04	10.50^#^	0.0012*
**Internal height (h_I_, cm)**	4.58±0.56	5.29±0.33	4.84^#^	0.028*
**External height (h_E_, cm)**	5.64±0.66	8.07±0.51	31.35∧	<0.0001*

Statistics include the mean ± 95% confidence interval, the F-ratio (∧) for parametric tests or Chi-square statistic/*X^2^* (^#^) for non-parametric tests, as well as the *P*-value.

*Indicates that there is a significant difference between the dimensions for each species (α = 0.05).

N = 8 for *Acanthagenys rufogularis* and N = 7 for *Manorina flavigula* except nest mass and density which has N = 3 and 7 (respectively). DF = 1,13 for all parametric comparisons except nest mass and density which has DF = 1,8. Non-parametric comparisons have a DF = 1. The replicate for the nest mass and density measurements is lower as some nests were excluded from analysis due to the attachment of supporting branches.

Surface area (Ā) is the geometric mean of the internal (A_I_) and external (A_E_) surface areas.

Of the eight *A. rufogularis* nests measured: four contained solid plant material such as stems, vines, rootlets and bark; five contained flat plant material such as leaves; all contained materials from graminoids such as grasses, sedges and rushes; all contained soft plant material (commonly known as plant down); five contained animal products such as wool, fur, hair and feathers; and all contained silk products from arachnids such as silk thread and egg sacs. Of the seven *M. flavigula* nests measured: all contained solid plant material; two contained flat plant material; six contained materials from graminoids; two contained soft plant material; all contained animal products and all contained silk products from arachnids. In addition, one nest contained man-made material (nylon). An indicator species analysis showed that the materials present in the nests did not differ between each species, with the exception of soft plant material (Table 3). Soft plant material occurred more often in *A. rufogularis* nests than in *M. flavigula* nests. Solid plant material was also found marginally more often in *M. flavigula* nests than in *A. rufogularis* nests, though the difference was not significant.

**Table 3:** Indicator species analysis output for materials found in *Acanthagenys rufogularis* and *Manorina flavigula* nests.

	Observed indicator value		Indicator value from randomised groups		
Material	*Acanthagenys rufogularis*	*Manorina flavigula*	Mean	Standard deviation	P-value
**Graminoids**	54	40	53.6	0.80	0.47
**Soft plant material**	78	6	49.9	8.41	0.0058*
**Solid plant material**	17	67	46.9	8.67	0.077
**Flat plant material**	43	9	36.1	10.39	0.31
**Animal products**	24	62	49.9	6.32	0.20
**Arachnid silk**	50	50	50.0	0.71	1.00

The indicator species analysis output includes the indicator values and Monte Carlo test of significance of observed maximum indicator values for materials found in *Acanthagenys rufogularis* and *Manorina flavigula* nests. Statistics include the observed indicator values (percent of perfect indication, based on combining relative abundance values and relative frequency) for nests of each species, mean and standard deviation for the indicator value from randomised groups, as well as the *P*-value. The *P*-value is the proportion of randomized trials with an indicator value equal to or exceeding the observed indicator value.

*Indicates that there is a significant correlation between the material type used in the nest and the species with the greater observed indicator value (α = 0.05).

N = 8 for *Acanthagenys rufogularis* and N = 7 for *Manorina flavigula*.

**Figure 5:**
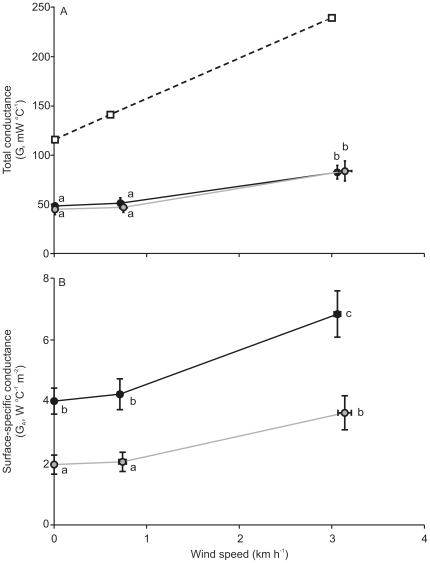
The effect of wind speed on nest conductance. Relationship between wind speed (km h^−1^) and (A) total conductance (G, mW °C^−1^) and (B) surface specific conductance (G_A_, W °C^−1^ m^−2^) for cup-shaped nests of *Acanthagenys rufogularis* (black line) and *Manorina flavigula* (grey line). The total conductance of the control (uncovered heat source) is also shown (dashed line). Each point represents the mean ± 95% CI for each treatment mean. Points that share common symbols (a,b,c) are those that are not significantly different according to a Tukey-Kramer HSD. Wind speed treatments consist of still, calm and light air.

There was a significant effect of wind speed on nest conductance for both species (Figure 5A, *X^2^* = 30.30, DF = 5, *P*<0.0001). There was no significant difference in the mean nest conductance for *A. rufogularis* and *M. flavigula*, within each wind speed treatment (Tukey-Kramer HSD). The control (unprotected heat source) had a conductance that was 250% greater than the nests of both species in still conditions and the difference increased to 290% in light air (Figure 5A); however the increase could not be analysed statistically as the control was not replicated. The conductance of the nest under the light air condition was greater than the still and calm conditions for both species; however the conductance was no greater in calm conditions than it was in still conditions for either *A. rufogularis* or *M. flavigula*.

There was a significant effect of wind speed on surface-specific nest conductance for *A. rufogularis* and *M. flavigula* (Figure 5B, *X^2^* = 37.19, DF = 5, *P*<0.0001). There was a significant difference in the mean surface-specific conductance for *A. rufogularis* and *M. flavigula*, within each wind speed treatment (Tukey-Kramer HSD). The surface-specific conductance of *A. rufogularis* nests was greater than *M. flavigula* for all three wind conditions. The surface-specific nest conductance in both species was significantly greater in light air than in calm or still conditions, which were not significantly different.

There was a significant effect of wind speed on thermal conductivity of the nest material for *A. rufogularis* and *M. flavigula* (Table 4, F_5,39_ = 27.90, *P*<0.0001). *M. flavigula* had a significantly greater mean thermal conductivity compared with *A. rufogularis*, within each wind speed treatment, as confirmed with the Tukey-Kramer HSD. The thermal conductivity under the light air condition was greater than the still and calm conditions for both species; however it was no greater in calm conditions than it was in still conditions for either *A. rufogularis* or *M. flavigula*.

**Table 4:** Comparison of the thermal and structural properties of nests of *Acanthagenys rufogularis* and *Manorina flavigula* under each wind speed treatment.

Species	Nest thickness(X)	Surface area(Ā)	Wind speed	Total conductance(G)	Surface-specific conductance(G_A_)	Thermal conductivity(k)
	cm	cm^2^		mW °C^−1^	W °C^−1^ m^−2^	mW °C^−1^ m^−1^
***Acanthagenys rufogularis***	0.93±0.19	122.46±12.08	**still**	48.4±2.7	4.0±0.4	36.7±6.0
			**calm**	51.3±5.3	4.2±0.5	38.7±6.8
			**light air**	82.7±6.8	6.8±0.8	63.0±11.8
***Manorina flavigula***	2.96±0.38	233.01±16.82	**still**	45.1±5.4	2.0±0.3	56.8±7.1
			**calm**	47.1±5.1	2.1±0.3	59.7±8.9
			**light air**	83.9±10.3	3.6±0.6	105.5±12.3

Statistics include the mean ± 95% confidence interval.

Surface area (Ā) is the geometric mean of the internal (A_I_) and external (A_E_) surface areas.

**Figure 6:**
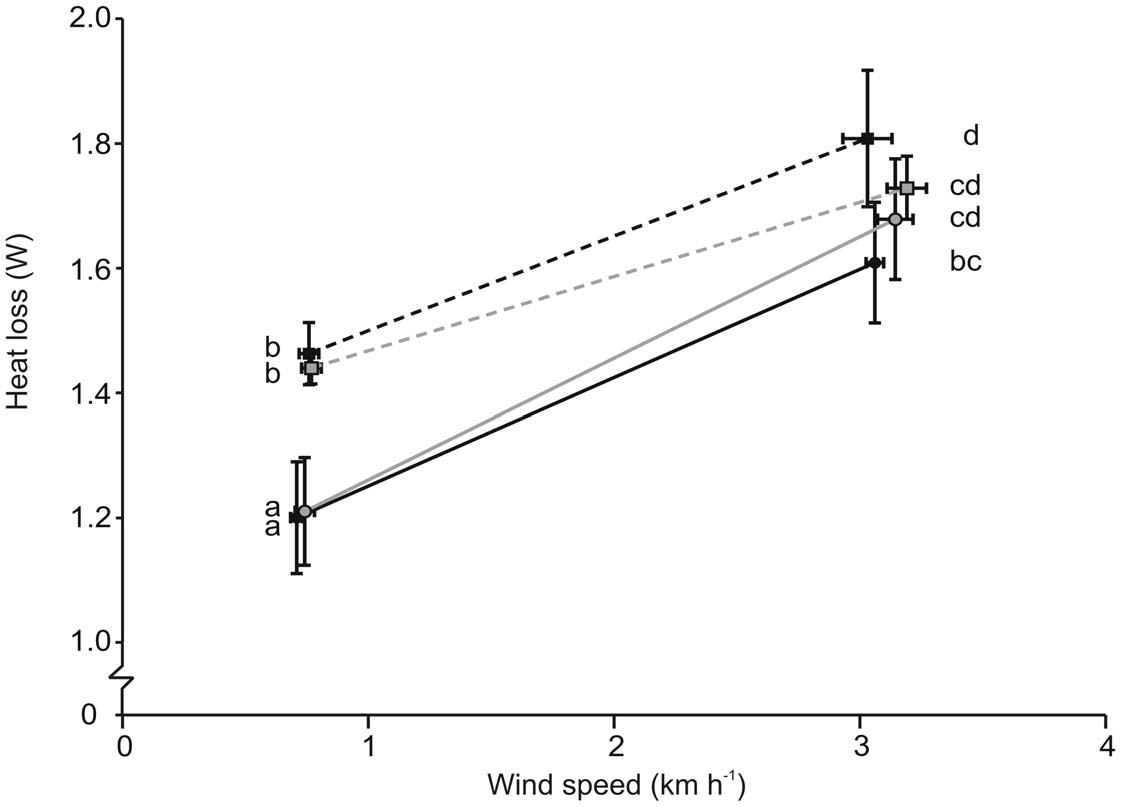
The effect of wind speed on heat loss from nests. Relationship between wind speed (km h^−1^) and heat loss (W) from cup-shaped nests of *Acanthagenys rufogularis* (black) and *Manorina flavigula* (grey) while nests are sealed (circles with solid lines) and open (square points with dashed lines). Each point represents the mean ± 95% CI for each treatment mean. Points that share common symbols (a,b,c,d) are those that are not significantly different according to a Tukey-Kramer HSD. Wind speed treatments consist of calm and light air.

There was a significant effect of wind speed on heat loss from the nests of *A. rufogularis* and *M. flavigula* when sealed and open (Figure 6, *X^2^* = 76.89, DF = 11, *P*<0.0001). There was a significant increase in the mean heat loss from *A. rufogularis* and *M. flavigula* as wind speed increased (Tukey-Kramer HSD). This was the case for both sealed and open nests. Heat loss from sealed *A. rufogularis* nests increased by 34% between the two wind speed treatments (calm and light air only), and by 24% from open nests. Heat loss from sealed *M. flavigula* nests increased by 39% between the two wind speed treatments and by 20% from open nests.

Within the calm treatment, there was no significant difference between the heat loss from *A. rufogularis* nests when open, compared with open *M. flavigula* nests. Nor was there a significant difference in the heat loss from sealed nests for either species. However, heat loss from the open nests was significantly higher than sealed nests for both species in calm conditions (22% higher for *A. rufogularis* and 19% for *M. flavigula*). Within the light air treatment, there was no significant difference in the heat loss from nests of *M. flavigula*, when sealed or open. However, open nests of *A. rufogularis* had a 12% greater rate of heat loss than sealed nests.

## Discussion

### (a) Still conditions

Nest conductance in still conditions is influenced by the thermal conductivity, the surface area and the thickness of the nest (Equation 2). The similarity in nest conductance between the two species results from a smaller surface area and lower thermal conductivity in *A. rufogularis* being compensated by a thinner nest (Table 4). *M. flavigula*, on the other hand, has a greater surface area and higher conductivity, but a much thicker nest. Given that the species have similar body masses and therefore comparable metabolic rates [43], the rate of heat loss from the nest appears independent of nest structure. The materials present in the nests do not differ between each species, with the exception of plant down, which is found more frequently in nests of *A. rufogularis* (Table 3) and consequently their thermal conductivity is lower (Table 4). Plant down is made up of many plant fibres such that it resembles fur and appears to be good insulation.

Despite differences in density (g cm^3^) between nests of the two species, density does not reflect the ability of the nest to prevent convective heat loss, but rather the fineness of weave is the important factor. The inner lining of the *M. flavigula* nest is densely woven but the main structure of the nest (the majority of the material) is quite loosely woven. On the other hand, the nest of *A. rufogularis* has a medium weave throughout, resulting in a greater overall nest density. This heterogeneous layered structure (Figure 1) means that is not possible in this study to compare heat loss from the nests in terms of the fineness of weave. Future work on convective heat loss from nests could assess nests that have a consistent structure throughout to determine if nest weave influences heat loss.

### (b) The effect of wind

i) **Sealed nests.** Light air flowing around the nest results in significant increases of between 71 to 86% in total conductance and surface-specific conductance in both species. The increase in conductance with wind speed is caused by disruption of the boundary layer around the nest and passage of air through it. The boundary layer is the still layer of air at the nest-environment interface [36].

Heat is transported through the boundary layer by conduction and is then transferred from the boundary layer largely by radiation and convection. The thickness of the boundary layer is a function of the wind velocity and the structure of the nest surface. For example, nests with a loose weave and plenty of sticks penetrating from the surface would have a thicker boundary layer than nests with a smooth weave. By increasing the wind speed around the nest, heat loss from the boundary layer is facilitated [44].

In addition, wind enters the nest material and convects heat away, as shown by the increase in the thermal conductivity with greater wind speeds (Table 4). While conductance is influenced by the nest dimensions and thermal conductivity, the dimensions of the nest do not change with increasing wind speed; hence the increase in conductance must be a result of the increase in convection through the nest. While the nest wall reduces the wind speed within the nest cup compared to the tunnel, wind speeds of 0.6 km h^−1^ and 0.8 km h^−1^ were detected in the nest in the light air condition for *A. rufogularis* and *M. flavigula*, respectively. The lower wind speed within *A. rufogularis* nests indicates that the nest wall provides more protection from the wind and may also contribute to the lower thermal conductivity of the nest in comparison to *M. flavigula*. It is also reasonable to assume that the wind speed within the nest increases with the wind speed in the tunnel, increasing the thermal conductivity at higher wind speeds.

The consequence of increased wind currents around and through the nests in these experiments would be a near-doubling in heat production required by the parent when incubating. There are clear energetic costs to some birds when wind speeds increase [15], [45] however convection is known to influence the choice of roosting site for non-breeding birds as well. Goldfinches (*Carduelis tristis*) save 12% of their energy by roosting in sheltered sites, whereas heat loss from the Carolina and mountain chickadees (*Parus carolinensis and Poecile gambeli*) is reduced by 38 to 100% when using sheltered sites, primarily a result of reduced exposure to wind [46]–[48]. Keep in mind, also, that birds nesting in the wild would be subjected to wind speeds greater than those measured here and therefore the effect of wind on conductance may be more pronounced in natural systems. In addition, wind has unpredictable fluctuations in speed and direction (turbulence) in natural systems, which was not replicated here [49]. It is likely that there is an ever-increasing effect of wind on convective heat loss from nests, which could considerably influence the incubation cost for the parent [44]. Further work on convective heat loss from birds' nests under stronger wind conditions should investigate this idea.

ii) **Open nests.** As the rate of heat loss from open nests is greater than for sealed nests in calm air, when an incubating bird takes leave to forage in calm conditions, the clutch will be subjected to rates of heat loss between 19 to 22% higher. This can increase developmental time and decrease hatching success [50]–[52]. When wind speed increases to light air, unsealed *A. rufogularis* nests lose 11% more heat than sealed nests under comparable wind conditions. However, the difference between sealed and open nests does not hold true for *M. flavigula* – the sealed nest loses heat at the same rate as the open nest. The disparity between the responses of the nests of these species may be related to the nest structure. The majority of *M. flavigula* nests have an uneven nest rim and when viewing them from the side, many small twigs can be seen extending above the nest lip horizon at an angle. This is rarely seen in the case of *A. rufogularis* nests. It may be that there is a greater opportunity for air leaks to occur in the sealed treatments for *M. flavigula*, compared to nests of *A. rufogularis*. The consequence of this may be that the rate of heat loss under light air conditions for *M. flavigula* nests appears more pronounced than it would be if an incubating parent was sitting on the nest, moulding their underbelly to the nest edges. Alternatively, the rate of heat loss from *M. flavigula* nests in light air may not increase when the lid is removed, as the coarse outer structure may help to break up the flow of air around the nest to deflect some of it away from the nest opening.

If the rates of heat loss between treatments accurately represent those of attended versus unattended nests, then it means that unattended *M. flavigula* clutches may not cool as fast as *A. rufogularis* clutches at increased wind speeds. This would have some bearing on the rates of nest attendance between the two species where *M. flavigula* could take longer recesses and forage for greater periods, in turn reducing the cost of reproduction for this species, as occurs in other species [53]–[56].

iii) **No nest.** The control treatment enables the nest data to be put into perspective. The conductance from the heat source with no protection from a nest (control) nearly tripled in light air conditions compared to when surrounded by a nest. This demonstrates that the nest does in fact help to ameliorate heat loss from the clutch by 58 to 65%, potentially reducing the energetic cost of incubation to the parent. Ar and Sidis [57] found that the nest of the blackbird (*Turdus merula*) helped reduce heat loss from eggs in still air by 25 to 30% however cooling times were halved when wind speed increased to 2.7 km h^−1^. In the present study, the difference in conductance from the uncovered heat source and the heat source protected by a nest increased with wind speed, suggesting that the presence of a nest becomes even more important as wind speed increases. Our findings provide further support for the benefit of selecting sheltered nest sites [15]. In addition to the nest itself, the surrounding vegetation can be important. This is the case for the song sparrow (*Melospiza melodia*), where nests are constructed on the lee side of the plant, such that surrounding shrubs reduce wind speed around the nest by 73% [58].

### (c) Summary

Here we have shown that in still air, the two species have a similar nest conductance, a result of differences in nest dimensions. The thickness of a nest is largely driven by the need to support the clutch and incubating parent when comparing over a broad range of bird sizes [35]. In addition, the size of the inner nest cup is somewhat determined by the size of incubating parent and clutch. Therefore, it could be argued that the overall construction of a nest is fixed to some extent. However, in habitats where there are extreme temperature changes, the nature of the nest may be essential to the development of the clutch and the efficiency of temperature regulation of the young [20], [59]. Therefore, the highly contrasting nest dimensions yet comparable conductance values may be indicative of the plasticity in nest design within a species, with the ultimate objective of achieving an appropriate nest microclimate. However, small birds (such as those studied here) may have more flexibility regarding structural design of nests than larger birds that may be more restricted [35]. Furthermore, as nest thickness increases with parent mass [35], the clutches of large birds may be protected from convection to a greater extent than those of small birds.

Exposing the sealed nests to greater wind speeds, raises nest conductance via a concurrent increase in the thermal conductivity. Contrary to predictions based on convective heat loss, conductance is similar in the two species, although they construct their nests in different parts of the canopy. *A. rufogularis* does not increase the insulative value of the nest to account for the poorer protection from wind afforded by its location near the ends of branches. *M. flavigula* nests would potentially have greater protection from the elements due to the dense canopy near the trunk, but the nest has a thick nest wall, further adding to the protection of the offspring.

The significance of convection through the nest is a near-doubling in heat production required by the parent when incubating in light air conditions. However, this may be more or less extreme for other species, depending on the ability of the nest to impede wind currents [17]. Heat is lost from nests more rapidly during parental recesses for both species at low wind speeds, with a minimal increase in heat loss for *A. rufogularis* in light air. This would result in an immediate increase in heat loss from the clutch, thereby lengthening development time and reducing hatching success or chick survival [52], [60], [61]. The incubating or brooding parent will also be affected following a recess as extra energy will be expended to rewarm the clutch [62], [63]. In conclusion, building a thermally favourable nest helps to save parental energy [13], however convection increases conductance of heat from the nest and therefore selecting an appropriate nest site that provides additional shelter is important for avian reproductive success.
